# Association Between Baseline Soluble ST2 Levels and Long-Term Outcomes After Percutaneous Coronary Intervention

**DOI:** 10.3390/medicina62081563

**Published:** 2026-08-14

**Authors:** Jaeho Byeon, Young Woo Song, Kyung Hoon Roh, Kyung An Kim, Soohyun Kim, Yeo Reum Kim, Ik Jun Choi

**Affiliations:** 1 Division of Cardiology, Department of Internal Medicine, Incheon St. Mary’s Hospital, College of Medicine, The Catholic University of Korea, Seoul 06591, Republic of Korea; teejh@naver.com (J.B.); syw5832@gmail.com (Y.W.S.); shto8209@naver.com (K.H.R.); kyungahn.kim@gmail.com (K.A.K.); tshibaka@naver.com (S.K.); jane9208@naver.com (Y.R.K.); 2 Catholic Research Institute for Intractable Cardiovascular Disease, College of Medicine, The Catholic University of Korea, Seoul 06591, Republic of Korea

**Keywords:** sST2, PCI, coronary artery disease, prognosis, mortality, biomarker

## Abstract

*Background and Objectives*: Soluble suppression of tumorigenicity-2 (sST2) is a biomarker associated with myocardial stress, inflammation, and fibrosis. Although elevated sST2 levels have demonstrated prognostic significance in heart failure and acute coronary syndromes, their clinical relevance in patients undergoing percutaneous coronary intervention (PCI) remains incompletely defined. This study aimed to evaluate the association between baseline serum sST2 levels and long-term clinical outcomes in an unselected real-world cohort of patients undergoing PCI. *Materials and Methods*: A total of 758 consecutive patients with coronary artery disease who underwent PCI between September 2015 and November 2017 were enrolled. Baseline serum sST2 levels were measured before PCI, and patients were stratified according to the median value (28.3 ng/mL). The primary endpoint was major adverse cardiac and cerebrovascular events (MACCEs), defined as a composite of cardiac death, nonfatal myocardial infarction, and nonfatal stroke. Multivariable Cox proportional hazards models and receiver operating characteristic analyses were performed to assess the association of sST2 with clinical outcomes and its discriminatory ability. *Results*: During a median follow-up of 28.3 months, MACCEs occurred in 19 patients (2.5%). Patients with higher sST2 levels had significantly higher rates of MACCEs (4.2% vs. 0.8%, *p* = 0.005) and all-cause mortality (11.9% vs. 2.6%, *p* < 0.001) than those with lower sST2 levels. Although the association between elevated sST2 and MACCE was attenuated after multivariable adjustment (adjusted hazard ratio, 3.28; 95% confidence interval, 0.91–11.83; *p* = 0.070), receiver operating characteristic analysis demonstrated moderate predictive performance for MACCE (AUC, 0.723) and all-cause mortality (AUC, 0.763). The optimal cutoff value for predicting MACCE was 52.2 ng/mL. *Conclusions*: Elevated baseline serum sST2 levels were associated with adverse long-term clinical outcomes in an unselected, real-world cohort of patients undergoing PCI. Further studies are warranted to clarify its prognostic role in this population.

## 1. Introduction

Soluble suppression of tumorigenicity-2 (sST2), a member of the interleukin-1 receptor family, is an established biomarker reflecting myocardial stress, inflammation, and fibrosis [1,2,3]. Elevated sST2 levels have been associated with adverse outcomes in patients with heart failure and acute coronary syndromes [4,5,6,7,8,9,10,11,12]. Experimental and clinical studies have suggested that sST2 is involved in myocardial injury, adverse ventricular remodeling, and systemic inflammatory activation, all of which are closely linked to the progression of cardiovascular disease [3,6,13,14].

Percutaneous coronary intervention (PCI) is widely performed for the treatment of coronary artery disease (CAD). Despite advances in revascularization strategies and guideline-directed medical therapy, patients undergoing PCI remain at substantial risk for subsequent adverse cardiovascular events. Therefore, identification of biomarkers that provide additional prognostic information may improve risk stratification and help identify high-risk patients.

Given its pathophysiological association with inflammation, myocardial stress, and fibrosis, sST2 has emerged as a potential prognostic biomarker in cardiovascular disease. However, the prognostic significance of baseline sST2 levels in patients with CAD undergoing PCI has not been fully established, particularly in real-world clinical practice.

Therefore, we sought to evaluate the association between baseline serum sST2 levels measured before PCI and long-term clinical outcomes, including major adverse cardiac and cerebrovascular events (MACCEs), in patients with CAD undergoing PCI.

## 2. Materials and Methods

### 2.1. Study Population

We initially identified 796 consecutive patients with CAD scheduled for PCI at Incheon St. Mary’s Hospital between September 2015 and November 2017. This study was designed as a real-world PCI registry; therefore, consecutive patients undergoing PCI were enrolled regardless of their clinical presentation, including both chronic coronary syndrome (CCS) and acute coronary syndrome (ACS), to reflect routine clinical practice. Patients with cardiogenic shock, end-stage renal disease requiring dialysis, insufficient blood samples, or those who did not undergo PCI were excluded.

**Figure 1:**
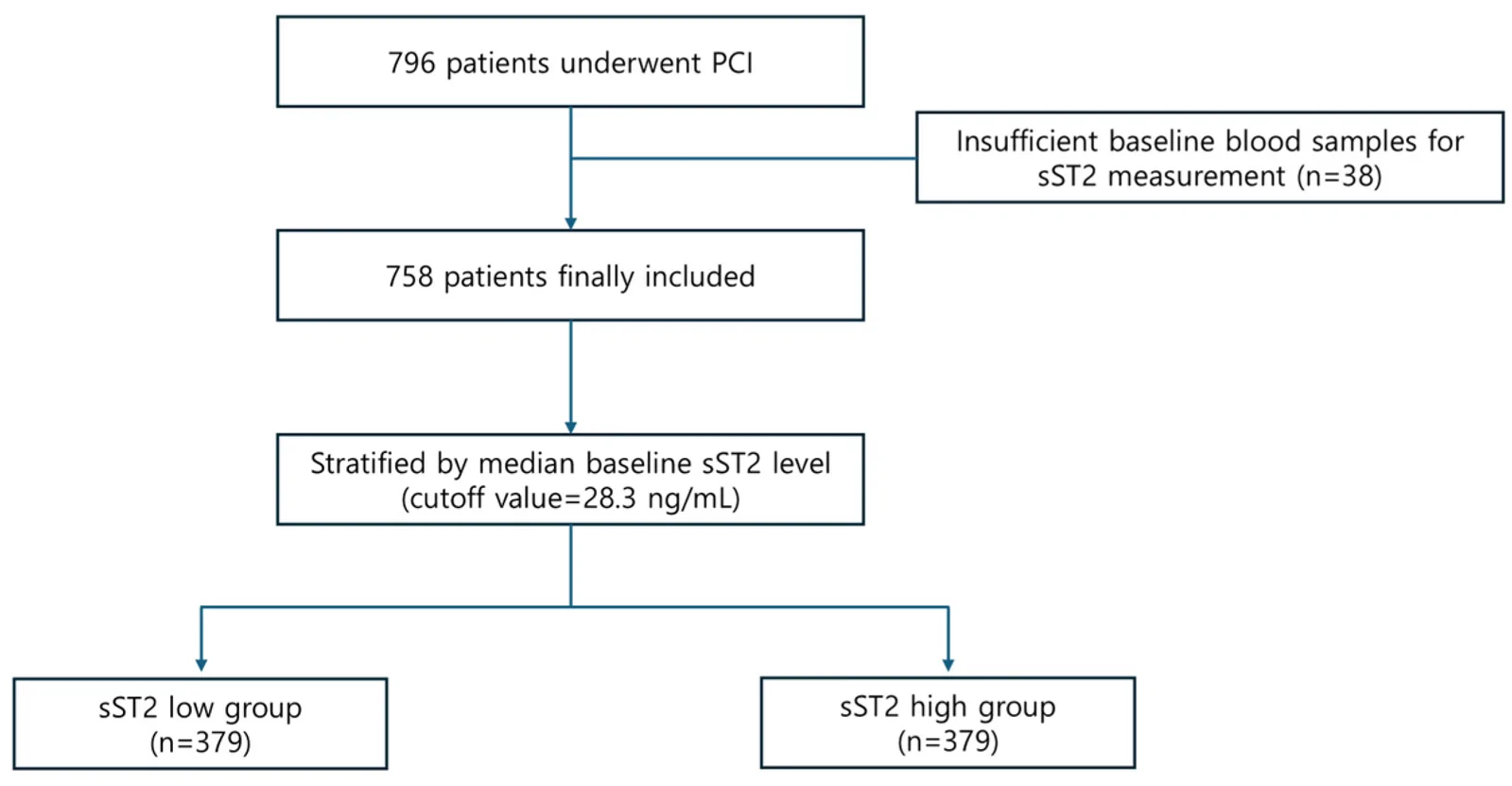
Flow diagram of the study population.

Among them, a total of 758 patients with available baseline serum samples for sST2 measurement before PCI were included in the final analysis (Figure 1). All participants provided written informed consent prior to PCI and blood sampling. The study protocol was approved by the Institutional Review Board of Incheon St. Mary’s Hospital (IRB No. OC15OISI0084) and complied with the Declaration of Helsinki.

### 2.2. PCI Procedure and Medical Treatment

Coronary angiography and PCI were performed using standard techniques at the discretion of the treating operator. All patients received standard antiplatelet therapy and periprocedural anticoagulation. Guideline-directed medical therapy, including antiplatelets, statins, beta-blockers, and renin–angiotensin–aldosterone system inhibitors, was administered according to the European and American guidelines prevailing at the time of patient enrollment [15,16]. Clinical follow-up was conducted at 3-month intervals after the index procedure.

### 2.3. Laboratory Measurements

Blood samples were obtained upon arrival at the catheterization laboratory, immediately after sheath insertion and prior to PCI. After centrifugation, serum samples were stored at −80 °C until analysis. Serum sST2 concentrations were measured using a commercially available enzyme-linked immunosorbent assay, the Presage ST2 Assay (Critical Diagnostics, San Diego, CA, USA), according to the manufacturer’s instructions. All measurements were performed by laboratory personnel blinded to clinical outcomes at the Clinical Research Laboratory of Incheon St. Mary’s Hospital.

### 2.4. Study Endpoints and Definitions

The primary endpoint was major adverse cardiac and cerebrovascular events (MACCEs), defined as a composite of cardiac death, nonfatal myocardial infarction, and nonfatal stroke. Clinical outcomes were assessed through hospital records and telephone interviews by trained investigators blinded to biomarker data. Mortality data were further verified using the National Health Insurance database. The secondary endpoints included the individual components of the primary endpoint, all-cause mortality, and any revascularization.

### 2.5. Statistical Analysis

Continuous variables are expressed as mean ± standard deviation and were compared using the independent *t*-test or Mann–Whitney U test, as appropriate. Categorical variables are presented as percentages and were compared using the chi-square test or Fisher’s exact test. Fisher’s exact test was additionally used when the expected event frequency was low.

Serum soluble ST2 concentrations were dichotomized according to the median value of the study population (28.3 ng/mL) into low and high sST2 groups, with the low sST2 group used as the reference group in all regression analyses. Because no universally accepted cutoff value for baseline sST2 has been established in unselected PCI populations, and previously reported thresholds vary across different clinical settings and study populations, the median value was prespecified as the primary categorization strategy to provide balanced group sizes for exploratory prognostic analyses. Receiver operating characteristic (ROC) curve analysis was additionally performed to determine the optimal cutoff value of sST2 for predicting clinical outcomes. Differences in baseline characteristics between the two groups were evaluated using the independent *t*-test or Mann–Whitney U test for continuous variables and the chi-square test or Fisher’s exact test for categorical variables.

Multivariable Cox proportional hazards models were used to assess the association between sST2 levels and clinical outcomes after adjustment for potential confounders, including age, sex, hemoglobin, high-sensitivity C-reactive protein (hs-CRP), and left ventricular ejection fraction. Hazard ratios (HRs) were calculated using the low sST2 group as the reference group. Subgroup and interaction analyses were performed to assess whether the association between sST2 and MACCEs differed between patients with CCS and ACS, using a multivariable Cox proportional hazards model with an interaction term between sST2 category and clinical presentation. Clinical presentation was not included as an additional covariate in the primary multivariable model because of the limited number of MACCEs. Missing data were infrequent for baseline covariates. Given the low proportion of missing data, no multiple imputation was performed, and multivariable Cox proportional hazards analyses were conducted using complete-case analysis. The proportional hazards assumption was evaluated using Schoenfeld residuals, and no significant violations were observed. Kaplan–Meier survival curves were generated, and differences between groups were assessed using the log-rank test. Restricted cubic spline analysis based on the Cox proportional hazards model was additionally performed to explore the potential nonlinear relationship between sST2 levels and clinical outcomes.

A two-sided *p*-value < 0.05 was considered statistically significant. Statistical analyses were performed using R version 4.2.1 (R Foundation for Statistical Computing, Vienna, Austria).

## 3. Results

### 3.1. Baseline Characteristics

Baseline characteristics of the study population are presented in Table 1. Among the 758 patients included in the analysis, the mean age was 65.9 ± 11.9 years, and 66.3% were male. Patients were divided into low and high sST2 groups according to the median baseline sST2 level (28.3 ng/mL). Compared with the low sST2 group, patients in the high sST2 group were older and had a lower body mass index, a higher prevalence of diabetes mellitus, lower left ventricular ejection fraction, higher levels of hs-CRP and NT-proBNP, and lower estimated glomerular filtration rate. In addition, patients in the high sST2 group more frequently presented with acute coronary syndrome, including unstable angina and NSTEMI, whereas stable angina was more common in the low sST2 group. The angiographic extent of coronary artery disease was generally similar between groups, with no significant difference in the prevalence of multivessel disease. Likewise, total stent number and mean stent diameter did not differ significantly according to sST2 status, although patients with higher sST2 levels underwent treatment with slightly longer total stent lengths.

### 3.2. Serum sST2 Levels and Clinical Outcomes

**Figure 2:**
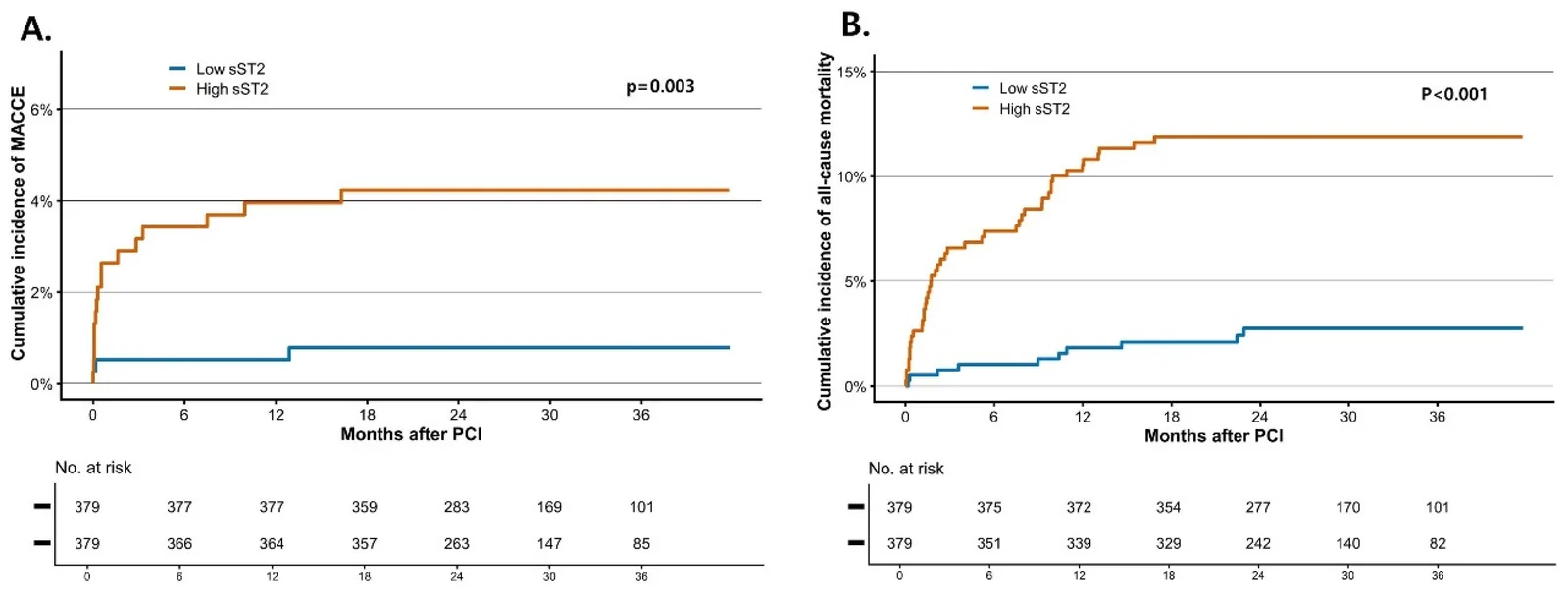
Kaplan–Meier curves according to baseline sST2 levels. (**A**) MACCE-free survival; (**B**) All-cause mortality.

During a median follow-up of 28.3 months (interquartile range, 23.7–35.9 months), MACCE occurred in 19 patients (2.5%). The incidence of MACCEs was significantly higher in the high sST2 group than in the low sST2 group (4.2% vs. 0.8%, *p* = 0.005; Table 2). Kaplan–Meier analysis demonstrated significantly lower MACCE-free survival in patients with high sST2 levels (Figure 2A; log-rank *p* = 0.003). Similarly, patients with high sST2 levels showed significantly lower overall survival and a higher incidence of all-cause mortality during follow-up compared with those with low sST2 levels (Figure 2B; log-rank *p* < 0.001). Kaplan–Meier analyses of the individual MACCE components, including cardiac death, myocardial infarction, and stroke, are provided in Supplementary Figure S1.

In univariate Cox regression analysis, high sST2 levels were significantly associated with an increased risk of MACCEs (hazard ratio [HR], 5.42; 95% confidence interval [CI], 1.58–18.59; *p* = 0.007; Table 3). In the multivariable Cox proportional hazards model adjusted for age, sex, left ventricular ejection fraction, hemoglobin, and high-sensitivity C-reactive protein, the high-sST2 group showed a numerically increased risk of MACCEs compared with the low-sST2 group; however, the association did not reach conventional statistical significance (adjusted HR, 3.28; 95% CI, 0.91–11.83; *p* = 0.070). In the exploratory subgroup analysis, the HRs for high versus low sST2 were 0.67 (95% CI, 0.06–7.48; *p* = 0.743) in patients with CCS and 4.96 (95% CI, 0.64–38.52; *p* = 0.126) in those with ACS, with no significant interaction (*p* for interaction = 0.212).

Patients with high sST2 levels also had significantly higher rates of all-cause mortality compared with those with low sST2 levels (11.9% vs. 2.6%, *p* < 0.001; Table 2). Cardiac death occurred more frequently in the high sST2 group (2.6% vs. 0.3%, *p* = 0.026). In contrast, the incidence rates of nonfatal myocardial infarction, nonfatal stroke, and any revascularization did not differ significantly between the groups.

### 3.3. ROC Analysis for sST2

Receiver operating characteristic (ROC) curve analysis was performed to evaluate the predictive performance of sST2 for MACCE and all-cause mortality. Baseline characteristics and clinical outcomes stratified according to the ROC-derived sST2 cutoff values are summarized in Supplementary Tables S1 and S2.

**Figure 3:**
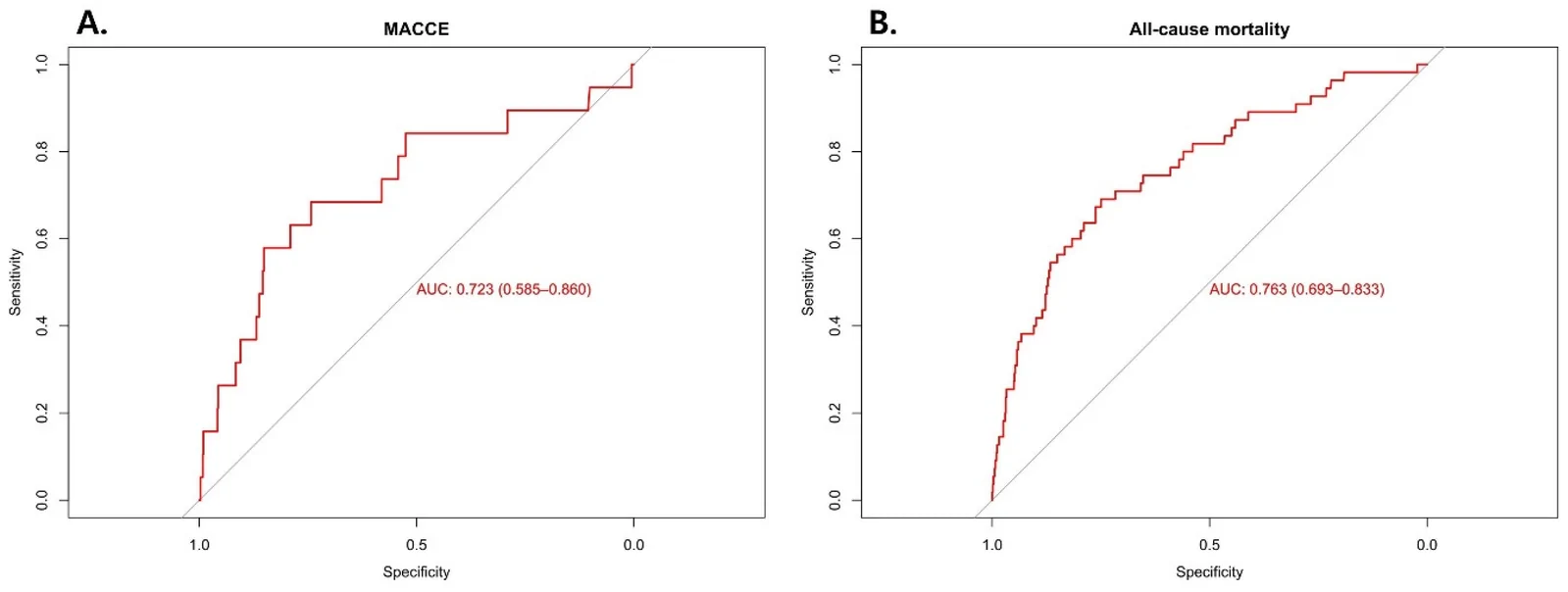
Receiver operating characteristic curves for baseline sST2 levels. (**A**) Prediction of MACCE; (**B**) Prediction of all-cause mortality.

ROC curve analysis for MACCE prediction is shown in Figure 3A. The area under the curve (AUC) was 0.723 (95% confidence interval [CI], 0.586–0.860), indicating moderate discriminatory ability. The optimal cutoff value determined by the Youden index was 52.2 ng/mL, with a sensitivity of 57.9% and a specificity of 85.1%.

Figure 3B presents the ROC curve analysis of sST2 for predicting all-cause mortality. The AUC was 0.763 (95% CI, 0.693–0.833), demonstrating moderate discriminatory performance. An optimal cutoff value of 37.6 ng/mL yielded a sensitivity of 69.1% and a specificity of 75.0%.

### 3.4. Restricted Cubic Spline Analysis of sST2 and Clinical Outcomes

**Figure 4:**
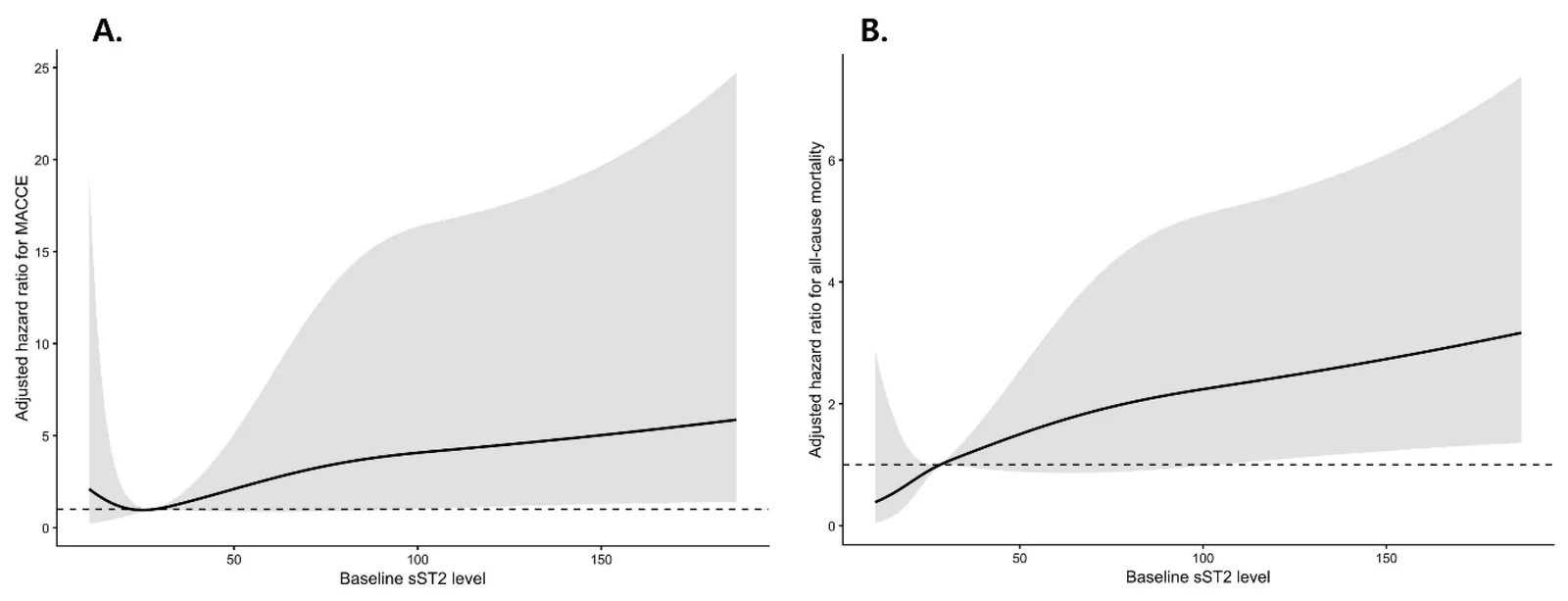
Restricted cubic spline analysis of baseline sST2 levels and clinical outcomes. Restricted cubic spline curves illustrating the associations between baseline sST2 levels and the risks of (**A**) MACCE and (**B**) all-cause mortality. Hazard ratios are shown relative to the reference value, with shaded areas representing 95% confidence intervals.

Restricted cubic spline analysis was performed to explore the relationship between baseline sST2 levels and clinical outcomes (Figure 4). For MACCE, increasing sST2 levels were associated with a numerically higher risk of adverse events, although the overall association did not reach statistical significance (overall *p* = 0.116). There was no significant evidence of a nonlinear relationship between sST2 levels and MACCE risk (nonlinearity *p* = 0.421).

In contrast, baseline sST2 levels were significantly associated with all-cause mortality (overall *p* = 0.012). The association appeared generally linear across the observed range of sST2 values, without significant evidence of nonlinearity (nonlinearity *p* = 0.255). These findings support a positive linear association between higher sST2 levels and mortality risk.

## 4. Discussion

In this real-world cohort of patients undergoing PCI, elevated baseline sST2 levels were associated with worse long-term clinical outcomes and higher mortality compared with lower baseline sST2 levels. Kaplan–Meier analysis also demonstrated significantly lower MACCE-free survival in patients with elevated sST2 levels. Although elevated sST2 was associated with a higher incidence of MACCEs in the unadjusted analyses, this association was attenuated after adjustment for relevant clinical covariates. Unlike previous studies that focused on selected CAD populations, our study evaluated an unselected real-world PCI cohort. Accordingly, the present findings address the prognostic relevance of baseline sST2 in patients undergoing PCI rather than the therapeutic effect of PCI itself.

Previous studies have consistently demonstrated the prognostic value of sST2 in patients with heart failure and acute coronary syndrome [9,11,17,18]. Recent evidence suggests that sST2 may also hold prognostic significance in patients with CAD undergoing PCI [3,19,20]. In a prospective cohort study by Li et al., elevated sST2 levels were independently associated with increased risks of long-term MACEs and all-cause mortality in patients with CAD [19]. Our findings extend these observations by demonstrating that elevated baseline sST2 levels were associated with adverse long-term outcomes in an unselected PCI cohort reflective of routine clinical practice. This association is biologically plausible, as sST2 serves as a decoy receptor that neutralizes cardioprotective IL-33 signaling, thereby promoting myocardial fibrosis, inflammation, and adverse ventricular remodeling [2,14].

An important finding of the present study is that the association between elevated sST2 levels and adverse clinical outcomes was observed despite a similar extent of angiographic coronary artery disease between groups, as the prevalence of multivessel disease did not significantly differ according to sST2 status. Previous studies have suggested that elevated sST2 reflects plaque vulnerability and inflammatory activation rather than anatomical coronary complexity alone. Luo et al. demonstrated that higher sST2 levels were associated with vulnerable plaque characteristics, including larger necrotic core volume and spotty calcification, while Demyanets et al. reported significantly higher sST2 levels in patients with ACS than in those with stable CAD [21,22]. In line with these findings, patients with elevated sST2 levels in our cohort more frequently presented with unstable angina and NSTEMI, despite having a similar angiographic burden of coronary artery disease. These observations suggest that sST2 may capture a biologically high-risk phenotype characterized by heightened inflammatory activity, plaque instability, and myocardial stress that is not fully reflected by conventional angiographic assessment alone. Exploratory subgroup analysis showed no statistically significant interaction between baseline sST2 category and clinical presentation (ACS versus CCS) for MACCE. However, only three MACCEs occurred in the CCS subgroup, resulting in wide confidence intervals and limited statistical power. Therefore, the absence of a significant interaction should not be interpreted as evidence of equivalent associations across ACS and CCS, and these findings should be interpreted cautiously.

Interestingly, the association between elevated sST2 levels and adverse outcomes appeared to be more strongly reflected in mortality outcomes than in recurrent ischemic events. This observation may reflect the broader biological role of sST2 as an integrated marker of systemic inflammation, myocardial stress, ventricular remodeling, and overall disease severity rather than a marker specific to recurrent coronary thrombosis alone.

ROC curve analysis demonstrated moderate discriminatory ability of sST2 for both MACCE and all-cause mortality, consistent with the observed association between elevated sST2 levels and adverse clinical outcomes. Similar findings have been reported in previous studies of patients with coronary artery disease, in which sST2 demonstrated modest but significant discriminatory ability for predicting adverse cardiovascular outcomes and mortality [19,20]. Collectively, these observations suggest that sST2 may serve as a useful biomarker when interpreted together with conventional clinical and laboratory risk parameters [19,22].

From a practical standpoint, baseline sST2 measurement before PCI may help identify patients at higher risk of adverse long-term clinical outcomes. Identification of these patients may facilitate closer surveillance, optimization of guideline-directed medical therapy, and more intensive secondary prevention strategies. Recent advances in guideline-directed medical therapy, particularly sodium-glucose cotransporter-2 (SGLT2) inhibitors, have changed the management of high-risk patients after myocardial infarction. In this context, biomarkers reflecting myocardial stress, such as sST2, may provide additional value for identifying patients who could benefit from optimized contemporary medical therapy [23]. In addition, incorporation of sST2 into existing clinical risk models may assist risk stratification alongside conventional angiographic and laboratory parameters. Future studies are warranted to determine whether incorporation of sST2 into established clinical risk models improves risk discrimination and clinical decision-making after PCI.

Several limitations should be acknowledged. First, this was a single-center observational study with a relatively limited number of clinical events, particularly MACCEs. Consequently, the multivariable analyses may have been underpowered and susceptible to model overfitting despite adjustment using prespecified clinically relevant covariates. This limited statistical power may have attenuated the association between baseline sST2 and MACCE after multivariable adjustment. Second, serum sST2 levels were measured only at baseline, and serial changes during follow-up were not evaluated. Third, although multivariable adjustment was performed, residual confounding cannot be completely excluded. Fourth, the optimal cutoff value of sST2 for risk stratification remains uncertain because sST2 levels may vary according to patient characteristics, clinical settings, and assay methods. Fifth, although the study included patients with diverse clinical presentations representative of routine PCI practice, the limited number of clinical events precluded definitive assessment of whether the association between baseline sST2 and clinical outcomes differed across clinical presentations. Finally, the study population consisted exclusively of Korean patients, which may limit the generalizability of the findings to other ethnic populations.

## 5. Conclusions

In conclusion, elevated baseline serum sST2 levels were associated with adverse long-term clinical outcomes in an unselected, real-world cohort of patients undergoing PCI. Further large prospective studies are warranted to clarify its prognostic role and determine whether sST2 provides incremental prognostic information beyond established clinical risk factors.

## Figures and Tables

**Table 1:** Baseline clinical and angiographic characteristics.

Variables	Overall (*n* = 758)	sST2 Low (*n* = 379)	sST2 High (*n* = 379)	*p* Value
Age (years)	65.9 ± 11.9	64.1 ± 11.1	67.8 ± 12.2	<0.001
Male	502 (66.3%)	249 (65.9%)	253 (66.8%)	0.857
Body mass index (kg/m^2^)	24.6 ± 3.9	25.2 ± 3.8	24.0 ± 3.9	<0.001
Hypertension	533 (70.4%)	257 (68.0%)	276 (72.8%)	0.168
Diabetes mellitus	290 (38.3%)	123 (32.5%)	167 (44.1%)	<0.001
Dyslipidemia	260 (34.3%)	139 (36.9%)	121 (32.5%)	0.241
Current smoking	224 (29.6%)	119 (31.5%)	105 (27.7%)	0.290
Family history of coronary artery disease	57 (7.5%)	33 (8.7%)	24 (6.3%)	0.266
Prior stroke	80 (10.6%)	34 (9.0%)	46 (12.1%)	0.198
Prior myocardial infarction	60 (7.9%)	31 (8.2%)	29 (7.7%)	0.885
Prior PCI	96 (12.7%)	51 (13.5%)	45 (11.9%)	0.576
Prior CABG	4 (0.5%)	1 (0.3%)	3 (0.8%)	0.618
Clinical presentation				<0.001
Stable angina pectoris	334 (44.1%)	222 (58.7%)	112 (29.6%)	
Unstable angina pectoris	239 (31.6%)	76 (20.1%)	163 (43.0%)	
NSTEMI	129 (17.0%)	48 (12.7%)	81 (21.4%)	
STEMI	14 (1.8%)	8 (2.1%)	6 (1.6%)	
Silent myocardial ischemia	35 (4.6%)	22 (5.8%)	13 (3.4%)	
LVEF (%)	52.3 ± 15.6	55.1 ± 15.3	49.5 ± 15.4	<0.001
High-sensitivity C-reactive protein (mg/L)	10.2 ± 28.0	3.9 ± 11.9	16.5 ± 36.7	<0.001
MDRD eGFR (mL/min/1.73 m^2^)	70.5 ± 29.8	74.9 ± 29.0	66.0 ± 30.0	<0.001
Hemoglobin (g/dL)	13.4 ± 2.5	13.7 ± 2.5	13.2 ± 2.4	0.005
NT-proBNP (pg/mL)	1912.5 ± 5859.7	644.2 ± 2891.6	3180.8 ± 7561.5	<0.001
Soluble ST2 (ng/mL)	39.0 ± 37.8	20.1 ± 4.7	57.8 ± 46.1	<0.001
Medications at discharge				
Aspirin	723 (95.5%)	369 (97.6%)	354 (93.4%)	0.009
Clopidogrel	459 (60.6%)	264 (69.8%)	195 (51.5%)	<0.001
Potent P2Y12 inhibitor	281 (37.1%)	110 (29.1%)	171 (45.1%)	<0.001
Statins	739 (97.6%)	373 (98.7%)	366 (96.6%)	0.104
Beta-blocker	523 (69.1%)	250 (66.1%)	273 (72.0%)	0.071
ACEi	351 (46.4%)	187 (49.5%)	164 (43.3%)	0.094
ARB	235 (31.0%)	120 (31.7%)	115 (30.3%)	0.695
Culprit coronary lesion				0.387
Left main	31 (4.1%)	19 (5.0%)	12 (3.2%)	
Left anterior descending artery	374 (49.3%)	198 (52.2%)	176 (46.4%)	
Left circumflex artery	126 (16.6%)	57 (15.0%)	69 (18.2%)	
Right coronary artery	198 (26.1%)	93 (24.5%)	105 (27.7%)	
Extent of CAD				0.059
1-vessel disease	317 (41.8%)	177 (46.7%)	140 (36.9%)	
2-vessel disease	229 (30.2%)	103 (27.2%)	126 (33.2%)	
3-vessel disease	130 (17.2%)	62 (17.9%)	68 (17.9%)	
Total stent number	1.3 ± 0.5	1.2 ± 0.5	1.3 ± 0.5	0.130
Mean stent diameter (mm)	3.1 ± 0.5	3.1 ± 0.5	3.1 ± 0.5	0.283
Total stent length (mm)	31.7 ± 18.1	30.1 ± 17.4	33.4 ± 18.6	0.014

Note: Values are presented as mean ± standard deviation or number (%). Percentages were calculated based on available data for each variable. Left main coronary artery disease was analyzed separately and was not included in the classification of 1-, 2-, or 3-vessel disease. Total stent number, mean stent diameter, and total stent length represent procedural characteristics of the culprit lesion treated during the index PCI. Abbreviations: sST2, soluble suppression of tumorigenicity-2; PCI, percutaneous coronary intervention; CABG, coronary artery bypass grafting; NSTEMI, non-ST-segment elevation myocardial infarction; STEMI, ST-segment elevation myocardial infarction; eGFR, estimated glomerular filtration rate; NT-proBNP, N-terminal pro-B-type natriuretic peptide; ACEi, angiotensin-converting enzyme inhibitor; ARB, angiotensin receptor blocker; CAD, coronary artery disease; LVEF, left ventricular ejection fraction.

**Table 2:** Clinical outcomes according to the level of sST2.

	sST2		HR (95% CI)	*p* Value
	Low	High		
MACCEs	3 (0.8%)	16 (4.2%)	5.42 (1.58–18.59)	0.007
All-cause mortality	10 (2.6%)	45 (11.9%)	4.75 (2.40–9.43)	<0.001
Cardiac death	1 (0.3%)	10 (2.6%)	Not estimated	0.026
Nonfatal MI	0 (0%)	2 (0.5%)	Not estimated	-
Nonfatal stroke	2 (0.5%)	4 (1.1%)	Not estimated	0.423
Any revascularization	7 (1.8%)	5 (1.3%)	Not estimated	0.586

Note: Values are presented as number (%). MACCE indicates a major adverse cardiac and cerebrovascular event, defined as cardiac death, myocardial infarction, or stroke. Abbreviations: HR indicates hazard ratio; CI, confidence interval; MI, myocardial infarction; sST2, soluble suppression of tumorigenicity-2.

**Table 3:** Predictors of MACCEs by univariate and multivariate Cox regression analyses.

	Univariate		Multivariate	
	HR (95% CI)	*p* Value	HR (95% CI)	*p* Value
Age	1.10 (1.04–1.15)	<0.001	1.07 (1.01–1.13)	0.022
Female	3.44 (1.36–8.74)	0.009	2.22 (0.80–6.22)	0.128
Body mass index	0.93 (0.86–1.01)	0.079		
Hypertension	2.24 (0.65–7.69)	0.200		
Diabetes mellitus	1.17 (0.47–2.91)	0.734		
Dyslipidemia	0.67 (0.24–1.85)	0.438		
Current smoker	0.28 (0.06–1.20)	0.086		
Family history of CAD	0.68 (0.09–5.13)	0.713		
Prior stroke	1.60 (0.47–5.50)	0.453		
Prior PCI	1.29 (0.38–4.43)	0.685		
Prior MI	2.20 (0.64–7.54)	0.211		
LVEF (%)	0.97 (0.95–0.99)	0.011	0.98 (0.96–1.01)	0.127
MDRD eGFR	0.98 (0.96–1.00)	0.072		
Hemoglobin	0.84 (0.73–0.98)	0.031	1.07 (0.86–1.33)	0.558
hs-CRP	1.01 (1.01–1.02)	<0.001	1.01 (1.00–1.01)	0.123
sST2 (per 10 ng/mL increase)	1.12 (1.06–1.17)	<0.001		
sST2 High	5.42 (1.58–18.59)	0.007	3.28 (0.91–11.83)	0.070

## Data Availability

The data presented in this study are available from the corresponding author upon reasonable request. The data are not publicly available due to privacy and ethical restrictions related to patient information.

## Supplementary Materials

The following supporting information can be downloaded at https://www.mdpi.com/article/10.3390/medicina62081563/s1, Figure S1: Kaplan–Meier analysis of baseline sST2 levels and individual clinical outcomes; Table S1: Baseline clinical and angiographic characteristics according to sST2 levels (cutoff value: 52.2 ng/mL); Table S2: Clinical outcomes according to the level of sST2 (cutoff value: 52.2 ng/mL).

## Abbreviations

The following abbreviations are used in this manuscript:

ACS	Acute coronary syndrome
AUC	Area under the curve
CAD	Coronary artery disease
CCS	Chronic coronary syndrome
CI	Confidence interval
ELISA	Enzyme-linked immunosorbent assay
eGFR	Estimated glomerular filtration rate
HR	Hazard ratio
hs-CRP	High-sensitivity C-reactive protein
IL	Interleukin
LVEF	Left ventricular ejection fraction
MACCE	Major adverse cardiac and cerebrovascular events
NSTEMI	Non-ST-segment elevation myocardial infarction
NT-proBNP	N-terminal pro-B-type natriuretic peptide
PCI	Percutaneous coronary intervention
ROC	Receiver operating characteristic
sST2	Soluble suppression of tumorigenicity-2
