# Supplementary material for: Association Between Baseline Soluble ST2 Levels and Long-Term Outcomes After Percutaneous Coronary Intervention
Source: Medicina (Kaunas). 2026 Aug 14;62(8):1563. doi: 10.3390/medicina62081563 (PMC13514982; doi:10.3390/medicina62081563)
Supplement: Supplementary file 1 [file medicina-62-01563-s001.zip › medicina-4423484-supplementary.pdf]

Supplementary data

Supplementary Figure S1. Kaplan–Meier Analysis of Baseline sST2 Levels and Individual Clinical Outcomes

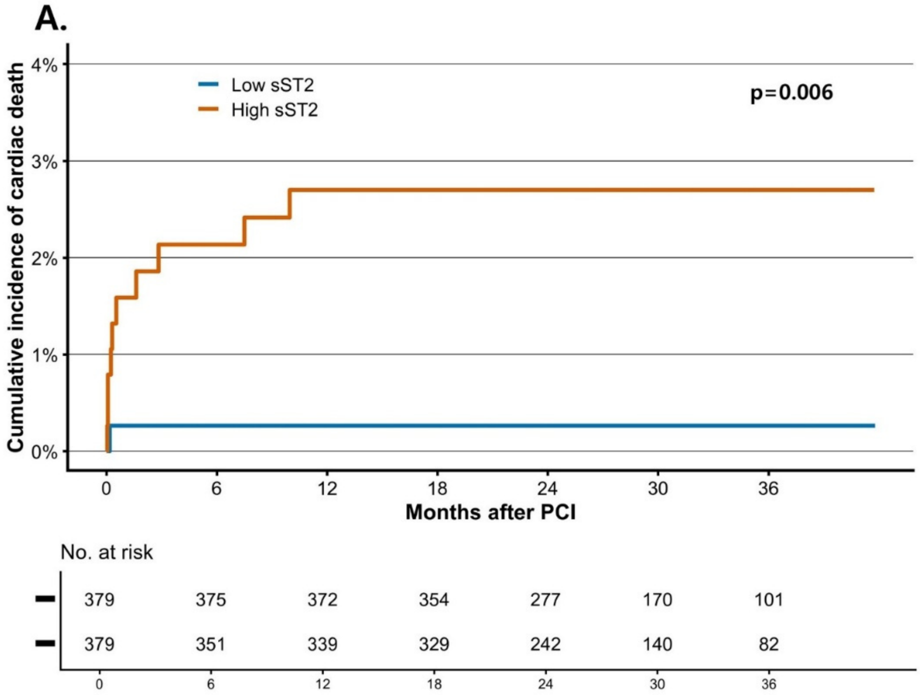

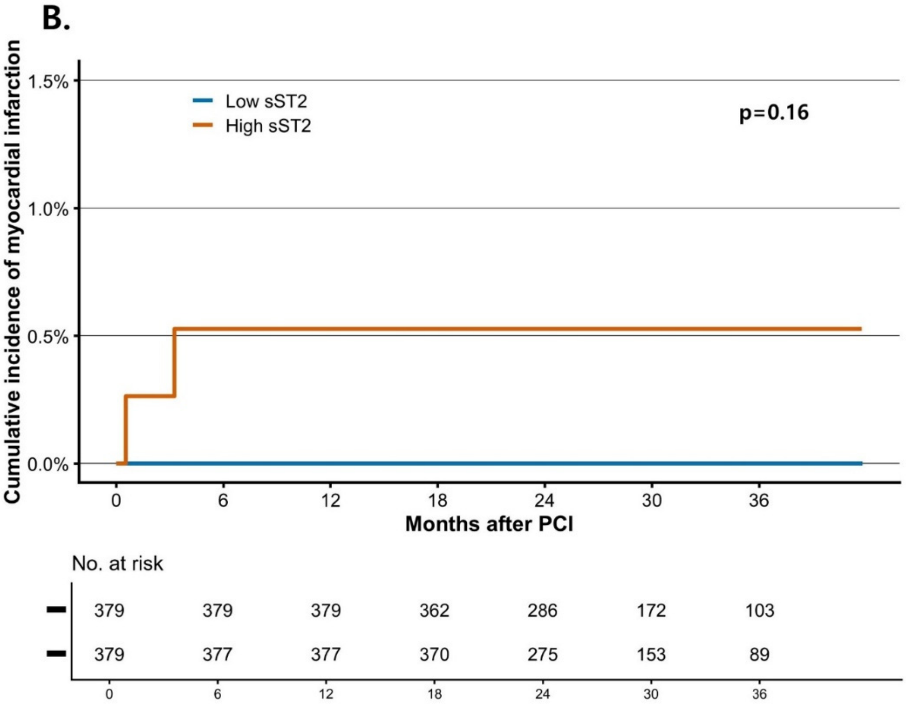

C.

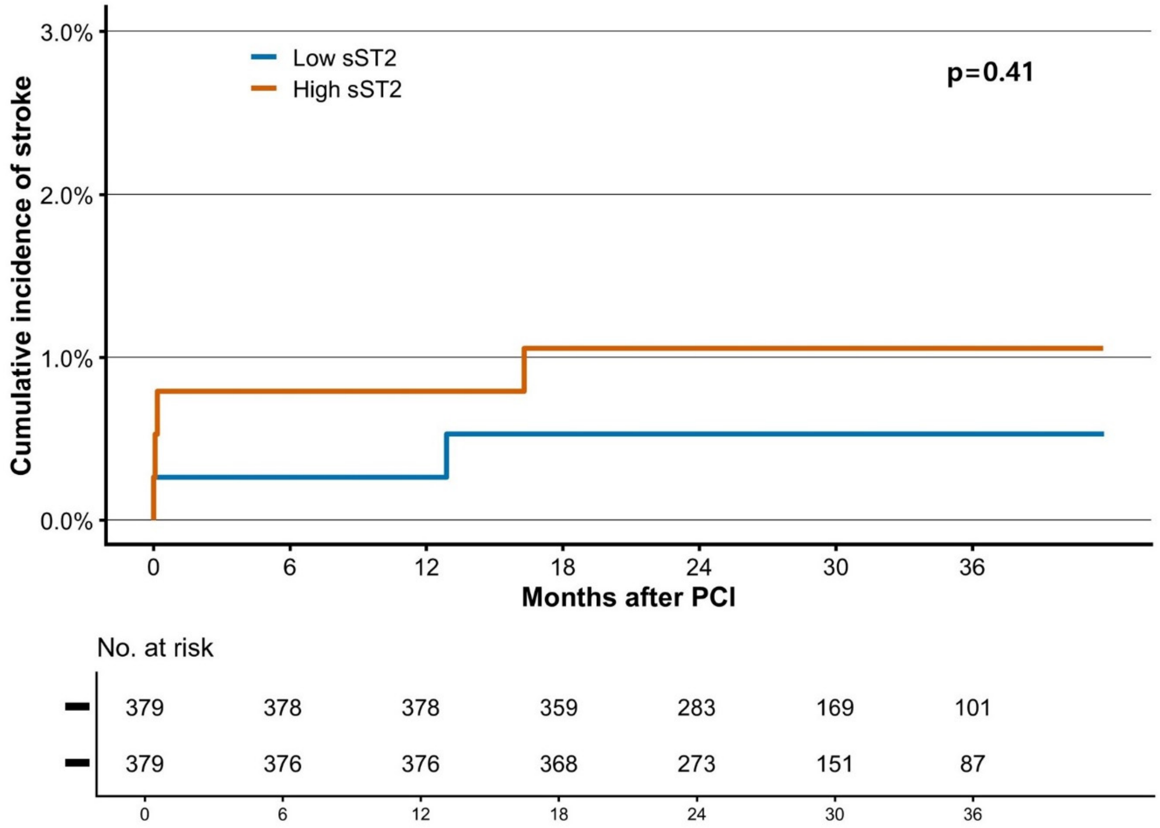

Kaplan–Meier curves showing the associations between baseline sST2 levels and the risks of (A) cardiac death, (B) myocardial infarction, and (C) stroke.

Supplementary Table S1. Baseline clinical and angiographic characteristics according to sST2 levels (cut-off value: 52.2 ng/mL)

Variables	sST2 <52.2 (n=637)	sST2 ≥52.2 (n=121)	*P value*
Age (years)	64.9 ± 11.6	71.6 ± 11.5	0.001
Male	425 (66.8%)	77 (63.6%)	0.565
Body mass index (kg/m^2)	24.9 ± 3.7	23.1 ± 4.4	0.001
Hypertension	442 (69.5%)	91 (75.2%)	0.249
Diabetes mellitus	231 (36.3%)	59 (48.8%)	0.013
Dyslipidemia	224 (35.5%)	36 (30.5%)	0.347
Current smoking	199 (31.3%)	25 (20.7%)	0.025
Family history of coronary artery disease	52 (8.2%)	5 (4.1%)	0.175
Prior stroke	63 (9.9%)	17 (14.0%)	0.231
Prior myocardial infarction	55 (8.6%)	5 (4.1%)	0.133
Prior PCI	88 (13.8%)	8 (6.6%)	0.041
Prior CABG	2 (0.3%)	2 (1.7%)	0.239
Clinical presentation			0.001
Stable angina pectoris	312 (49.1%)	22 (18.2%)	
Unstable angina pectoris	178 (28.0%)	61 (50.4%)	
NSTEMI	99 (15.6%)	30 (24.8%)	
STEMI	11 (1.7%)	3 (2.5%)	
Silent myocardial ischemia	33 (5.2%)	2 (1.7%)	
LVEF (%)	53.9 ± 15.0	44.0 ± 16.2	<0.001
hs-CRP (mg/l)	5.2 ± 14.5	36.5 ± 54.8	<0.001
MDRD eGFR (mL/min/1.73m^2)	73.2 ± 29.7	56.3 ± 26.6	<0.001
Hemoglobin (g/dL)	13.6 ± 2.4	12.6 ± 2.7	<0.001
NT-proBNP (pg/mL)	966.1 ± 3594.8	6912.9 ± 10887.7	<0.001
Soluble ST2 (ng/mL)	26.8 ± 9.8	103.0 ± 59.9	<0.001
Medications at discharge			

Aspirin	620 (97.5%)	103 (85.1%)	<0.001
Clopidogrel	406 (63.8%)	53 (43.8%)	<0.001
Potent P2Y12 inhibitor	223 (35.1%)	58 (47.9%)	0.008
Statins	628 (98.7%)	111 (91.7%)	<0.001
Beta-blocker	444 (69.8%)	79 (65.3%)	0.337
ACEi	300 (47.2%)	51 (42.1%)	0.322
ARB	213 (33.5%)	22 (18.2%)	0.001
Culprit coronary lesion			0.095
Left main	28 (4.4%)	3 (2.5%)	
Left anterior descending artery	320 (50.2%)	54 (44.6%)	
Left circumflex artery	99 (15.5%)	27 (22.3%)	
Right coronary artery	170 (26.7%)	28 (23.1%)	
Extent of CAD			0.231
1-vessel disease	274 (43.0%)	43 (35.5%)	
2-vessel disease	188 (29.5%)	41 (33.9%)	
3-vessel disease	110 (17.3%)	20 (16.5%)	
Total stent number	1.2 ± 0.5	1.3 ± 0.5	0.224
Mean stent diameter	3.1 ± 0.5	3.0 ± 0.5	0.011
Total stent length	31.6 ± 18.0	32.4 ± 18.7	0.696

Values are presented as mean ± standard deviation or number (%).
Percentages were calculated based on available data for each variable.
Left main coronary artery disease was analyzed separately and was not included in the classification of 1-, 2-, or 3-vessel disease.
Total stent number, mean stent diameter, and total stent length represent procedural characteristics of the culprit lesion treated during the index PCI.

Abbreviations: sST2, soluble suppression of tumorigenicity-2;
PCI, percutaneous coronary intervention;
CABG, coronary artery bypass grafting;
NSTEMI, non–ST-segment elevation myocardial infarction;
STEMI, ST-segment elevation myocardial infarction;
eGFR, estimated glomerular filtration rate;
NT-proBNP, N-terminal pro–B-type natriuretic peptide;

ACEi, angiotensin-converting enzyme inhibitor;
ARB, angiotensin receptor blocker;
CAD, coronary artery disease;
LVEF, left ventricular ejection fraction.

Supplementary Table S2. Clinical outcomes according to the level of sST2 (cut-off value: 52.2 ng/mL)

	sST2 <52.2	sST2 ≥52.2	HR (95% CI)	p value
MACCE	8 (1.3%)	11 (9.1%)	7.55 (3.04-18.77)	< 0.001
All-cause mortality	26 (4.1%)	29 (24%)	6.77 (3.99-11.5)	< 0.001
Cardiac death	2 (0.3%)	9 (7.4%)	Not estimated	< 0.001
Nonfatal MI	1 (0.2%)	1 (0.2%)	Not estimated	0.240
Nonfatal stroke	5 (0.8%)	1 (0.8%)	Not estimated	0.961
Any revascularization	10 (1.6%)	2 (1.7%)	Not estimated	0.931

Values are presented as number (%).

MACCE indicate major adverse cardiac and cerebrovascular events, defined as cardiac death, myocardial infarction, or stroke.

HR indicates hazard ratio;
CI, confidence interval;
MI, myocardial infarction;
sST2, soluble suppression of tumorigenicity-2.
